# Supplementary figures and images for: Moderation effects of food intake on the relationship between urinary microbiota and urinary interleukin-8 in female type 2 diabetic patients
Source: PeerJ. 2020 Jan 28;8:e8481. doi: 10.7717/peerj.8481 (PMC6993747; doi:10.7717/peerj.8481)

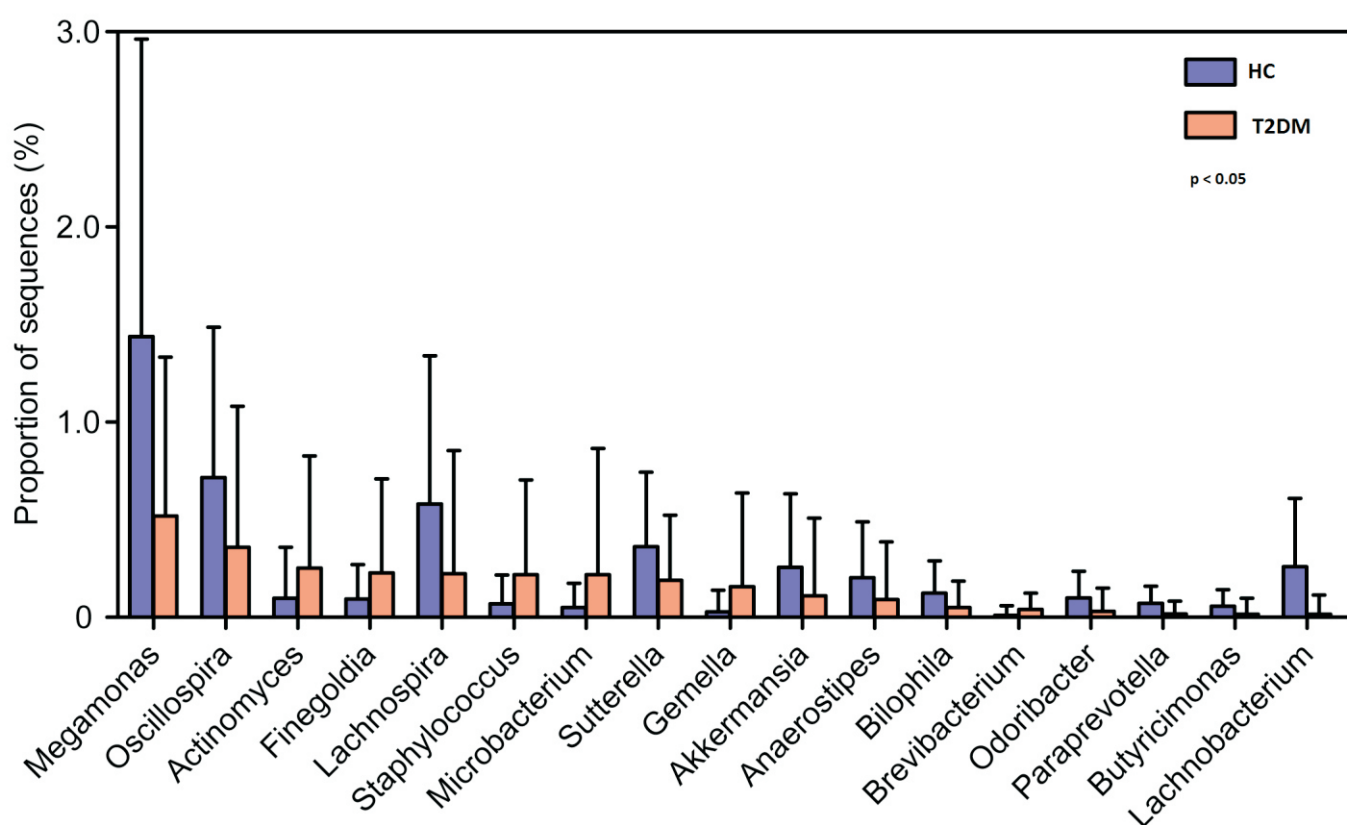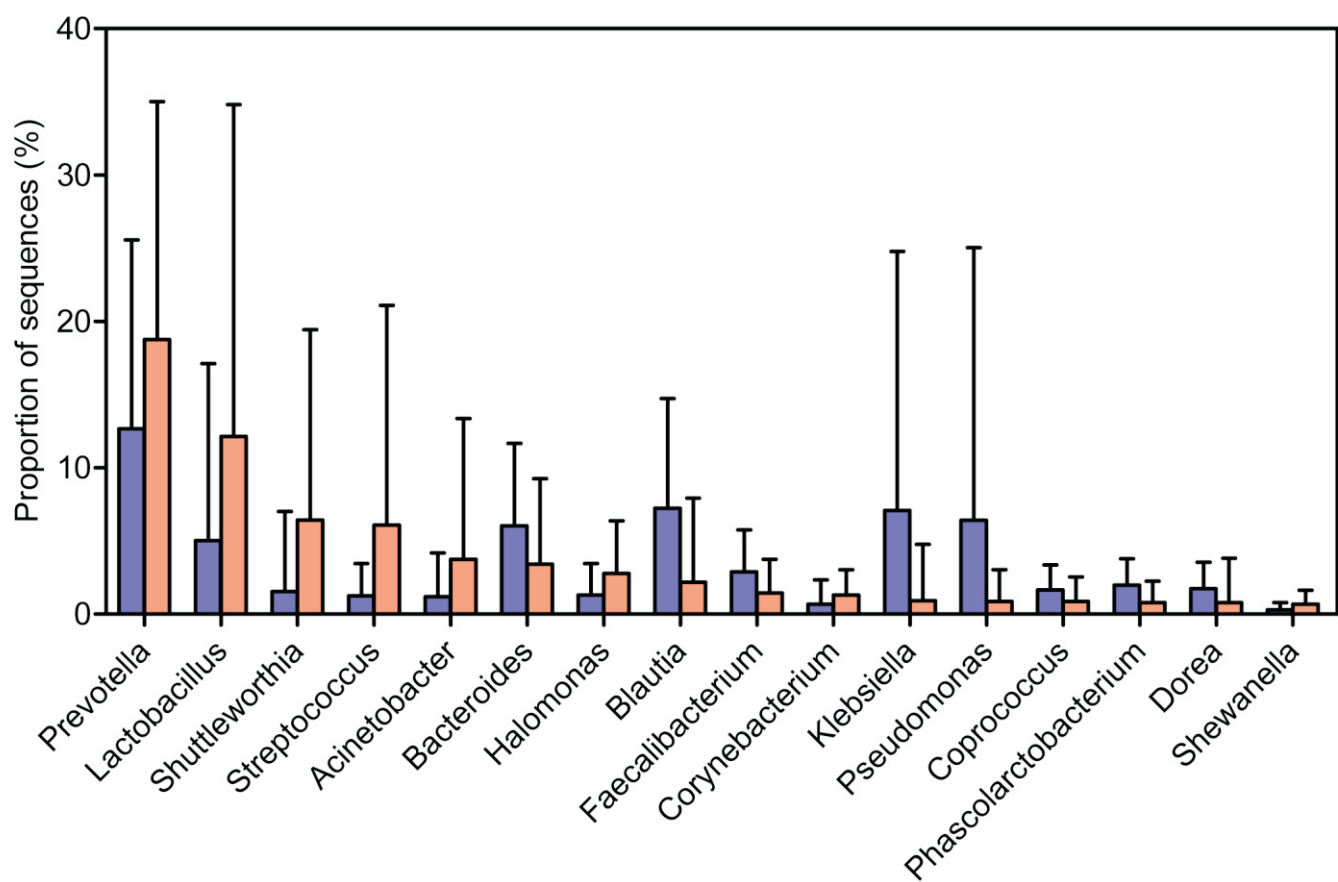

Supplement: Supplemental Information 1 [file peerj-08-8481-s001.pdf]

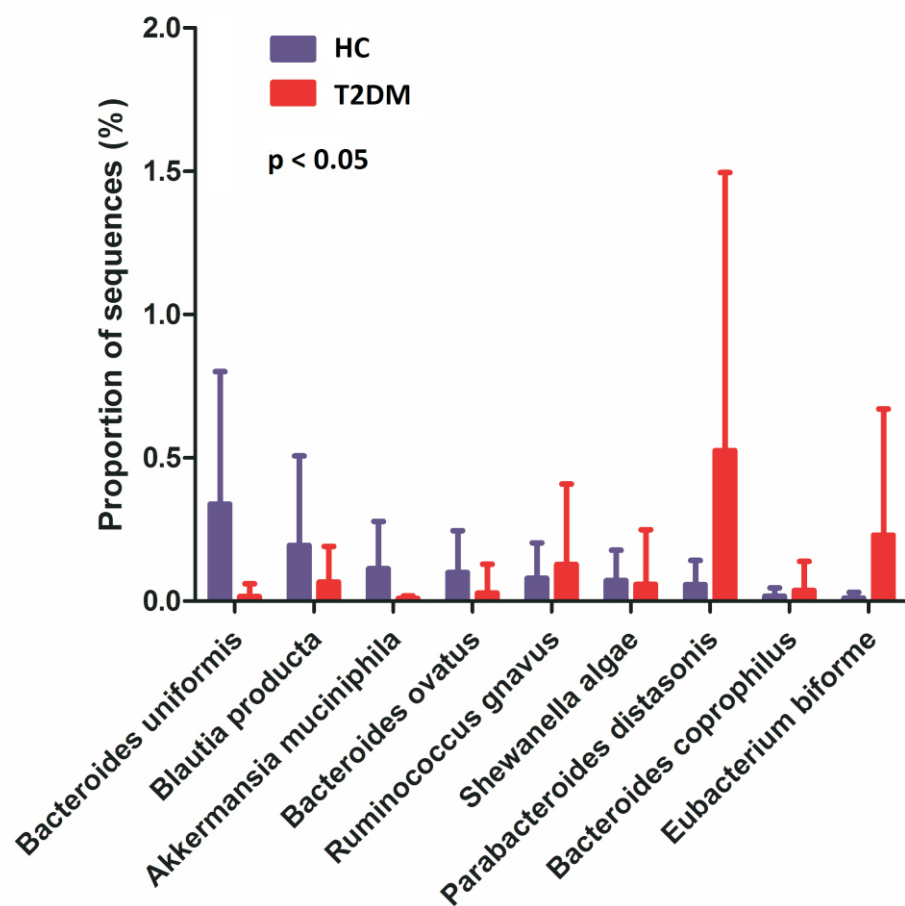

Supplement: Supplemental Information 2 [file peerj-08-8481-s002.pdf]

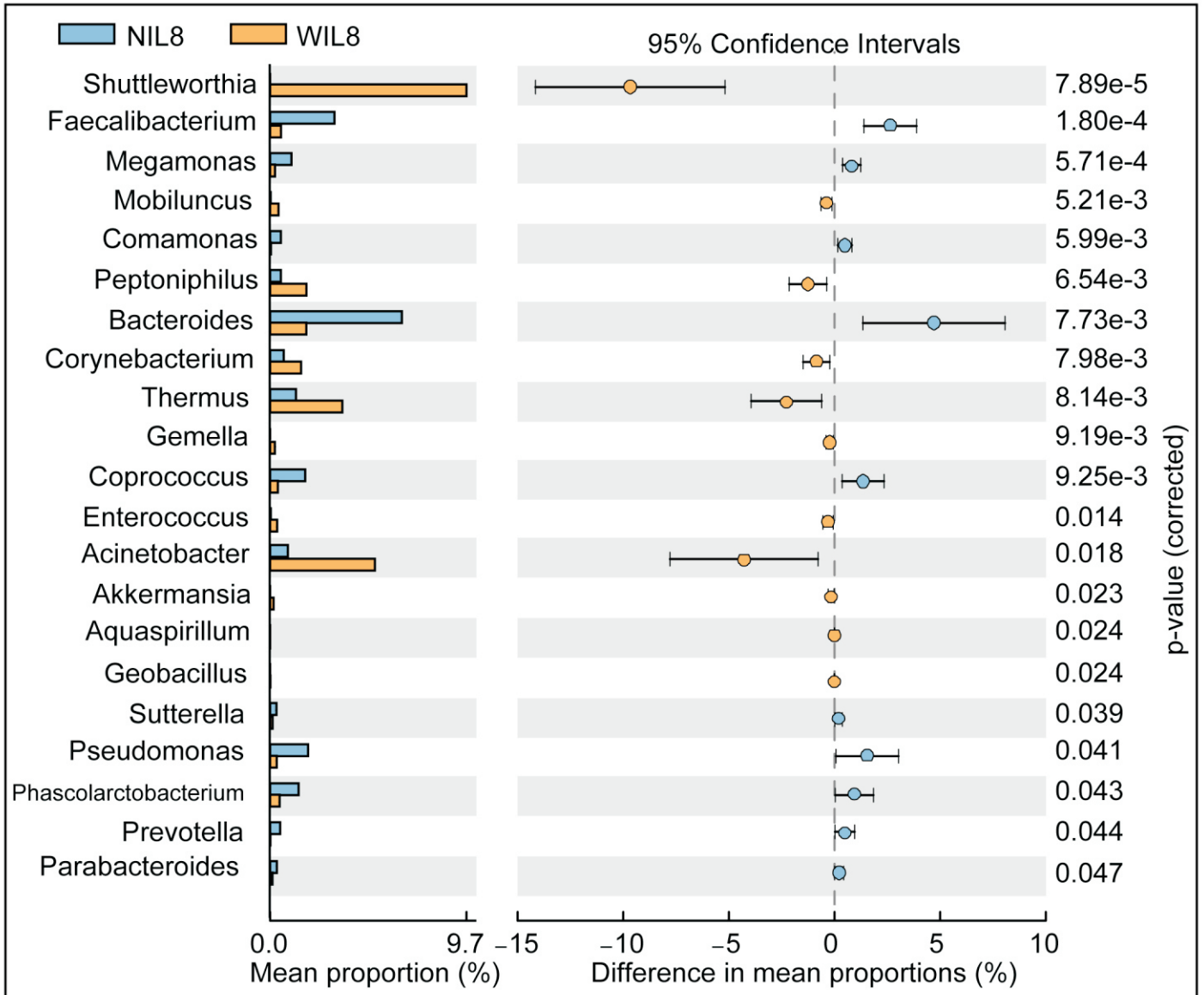

Supplement: Supplemental Information 3 [file peerj-08-8481-s003.pdf]

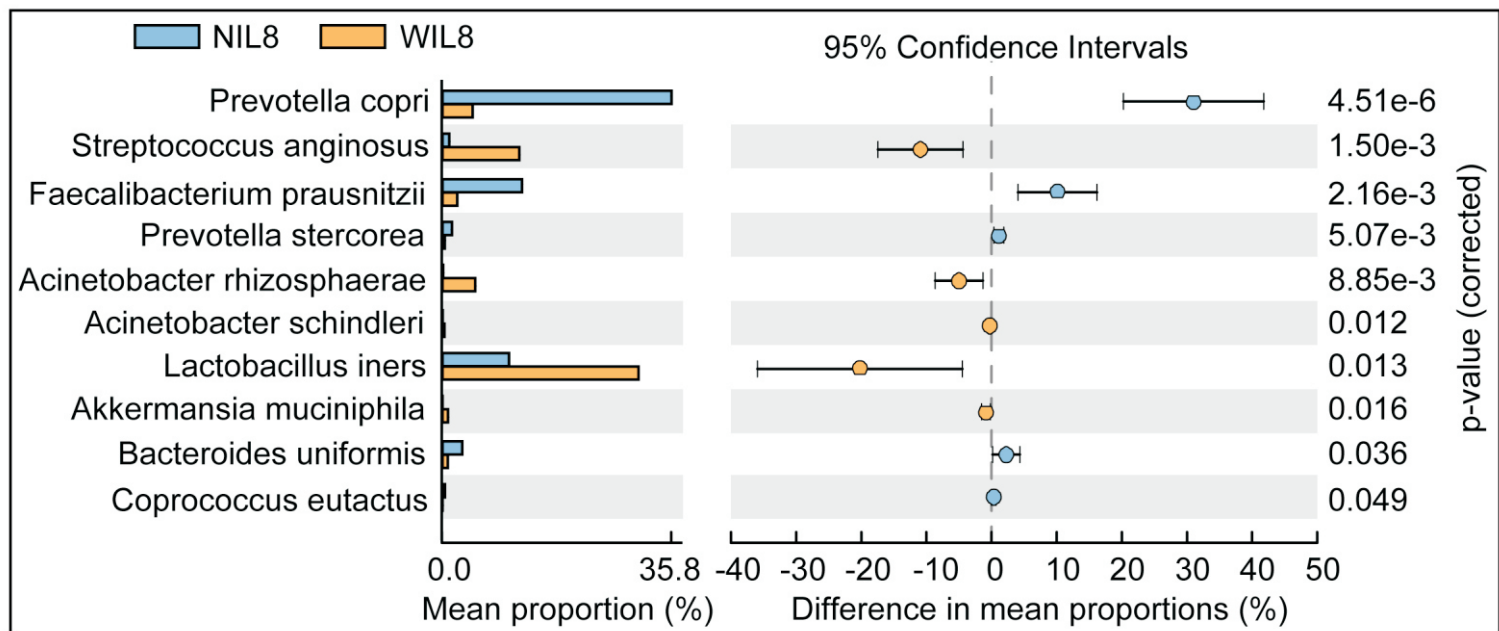

Supplement: Supplemental Information 4 [file peerj-08-8481-s004.pdf]

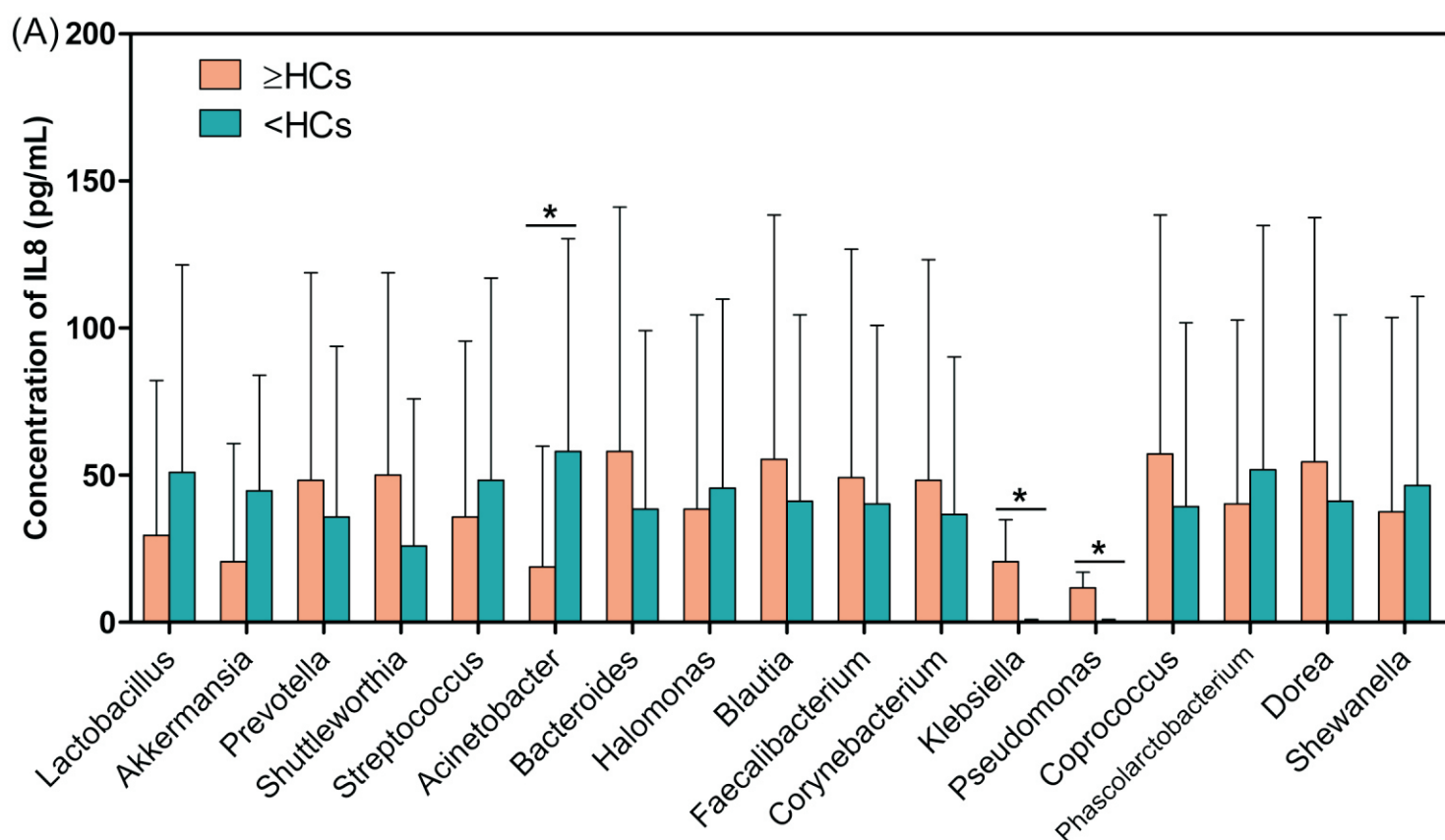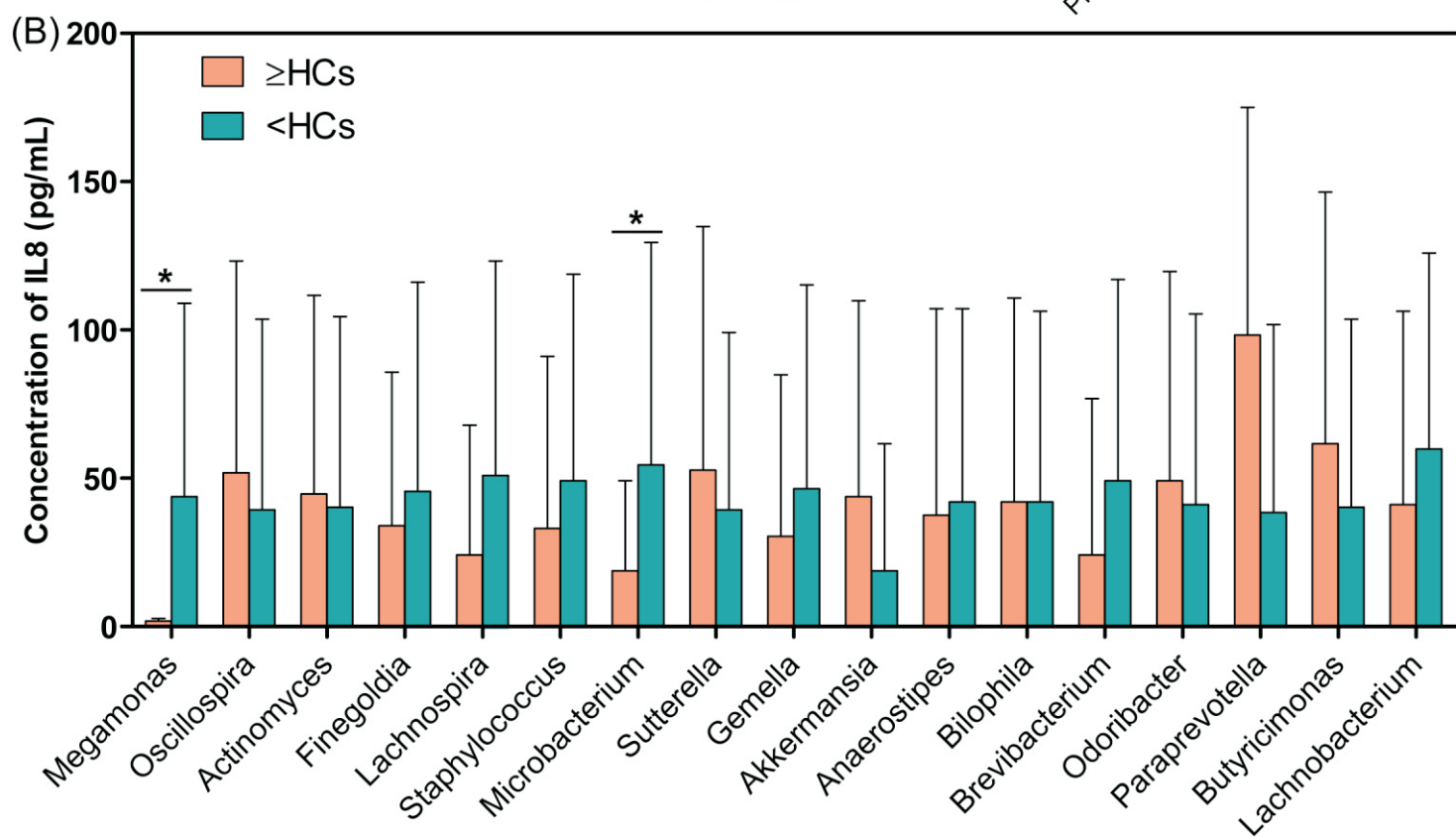

Supplement: Supplemental Information 5 [file peerj-08-8481-s005.pdf]
