# Supplementary material for: Moderation effects of food intake on the relationship between urinary microbiota and urinary interleukin-8 in female type 2 diabetic patients
Source: PeerJ. 2020 Jan 28;8:e8481. doi: 10.7717/peerj.8481 (PMC6993747; doi:10.7717/peerj.8481)
Supplement: Supplemental Information 6 [file peerj-08-8481-s006.pdf]

Table S1 Predictors of urine IL8 by stepwise regression (n=70)

Independent variables	Unstandardized coefficient		Standardized coefficient	t	p-value	F	p-value
	B	SE	β				
Constant	7.61	3.204		2.375	0.021	57.478	.000
Actinomyces	24.412	4.197	0.205	5.816	0.000		
Anaerotruncus	800.311	71.947	0.333	11.124	0.000		
Cloacibacterium	-76.561	23.057	-0.105	-3.32	0.002		
Cytophaga	4204.23	330.817	0.481	12.709	0.000		
Dokdonella	-1478.647	617.393	-0.075	-2.395	0.02		
Dysgonomonas	5080.332	1180.355	0.144	4.304	0.000		
Exiguobacterium	-258.281	67.919	-0.15	-3.803	0.000		
Flavisolibacter	-105.62	24.71	-0.155	-4.274	0.000		
Giesbergeria	296.428	29.784	0.355	9.953	0.000		
Lactobacillus	0.304	0.092	0.107	3.292	0.002		
Limnohabitans	4242.295	353.48	0.36	12.002	0.000		
Luteibacter	2307.744	347.269	0.199	6.645	0.000		
Meiothermus	20.241	2.496	0.249	8.11	0.000		
Providencia	13214.688	1239.622	0.319	10.66	0.000		
Ruminococcus	9.051	0.749	0.38	12.085	0.000		
Solitalea	849.692	74.052	0.352	11.474	0.000		
Ureaplasma	13.178	2.615	0.162	5.039	0.000		
Zoogloea	-3614.663	807.044	-0.147	-4.479	0.000		
