# Supplementary material for: Moderation effects of food intake on the relationship between urinary microbiota and urinary interleukin-8 in female type 2 diabetic patients
Source: PeerJ. 2020 Jan 28;8:e8481. doi: 10.7717/peerj.8481 (PMC6993747; doi:10.7717/peerj.8481)
Supplement: Supplemental Information 7 [file peerj-08-8481-s007.docx]

**Table S2 Relative abundance of bacteria involved in hierarchical regression models**

| **Taxon** | **Mean ± SD** |
| --- | --- |
| *Acinetobacter* | 5.460 ± 11.557 |
| *Acinetobacter rhizosphaerae* | 3.538 ± 10.716 |
| *Acinetobacter schindleri* | 0.219 ± 0.564 |
| *Actinomyces* | 0.210 ± 0.464 |
| *Akkermansia* | 0.060 ± 0.210 |
| *Akkermansia muciniphila* | 0.585 ± 1.989 |
| *Anaerotruncus* | 0.002 ± 0.017 |
| *Aquaspirillum* | 0.000 ± 0.016 |
| *Bacteroides* | 4.710 ± 8.634 |
| *Bacteroides coprophilus* | 0.003 ± 0.015 |
| *Bacteroides ovatus* | 0.037 ± 0.102 |
| *Bacteroides uniformis* | 1.539 ± 3.049 |
| *Blautia producta* | 0.058 ± 0.193 |
| *Cloacibacterium* | 0.043 ± 0.083 |
| *Comamonas* | 0.280 ± 0.810 |
| *Coprococcus* | 0.700 ± 1.551 |
| *Coprococcus eutactus* | 0.128 ± 0.472 |
| *Corynebacterium* | 1.290 ± 1.639 |
| *Cytophaga* | 0.001 ± 0.007 |
| *Dokdonella* | 0.000 ± 0.014 |
| *Dysgonomonas* | 0.000 ± 0.001 |
| *Enterococcus* | 0.340 ± 0.936 |
| *Eubacterium biforme* | 0.002 ± 0.008 |
| *Exiguobacterium* | 0.030 ± 0.104 |
| *Faecalibacterium* | 1.520 ± 2.668 |
| *Faecalibacterium prausnitzii* | 5.488 ± 9.445 |
| *Gemella* | 0.170 ± 0.503 |
| *Geobacillus* | 0.090 ± 0.526 |
| *Giesbergeria* | 0.009 ± 0.054 |
| *Klebsiella* | 0.280 ± 0.964 |
| *Lactobacillus* | 12.450 ± 22.336 |
| *Lactobacillus iners* | 24.260 ± 32.584 |
| *Limnohabitans* | 0.000 ± 0.004 |
| *Luteibacter* | 0.000 ± 0.004 |
| *Megamonas* | 0.800 ± 1.368 |
| *Meiothermus* | 0.060 ± 0.402 |
| *Microbacterium* | 0.050 ± 0.133 |
| *Mobiluncus* | 0.220 ± 0.615 |
| *Parabacteroides* | 0.330 ± 0.593 |
| *Parabacteroides distasonis* | 0.027 ± 0.073 |
| *Peptoniphilus* | 2.130 ± 3.640 |
| *Prevotella* | 18.450 ± 14.783 |
| *Prevotella copri* | 14.475 ± 20.514 |
| *Prevotella stercorea* | 0.710 ± 1.252 |
| *Providencia* | 0.000 ± 0.002 |
| *Pseudomonas* | 1.510 ± 4.545 |
| *Ruminococcus* | 1.250 ± 2.866 |
| *Ruminococcus gnavus* | 0.016 ± 0.045 |
| *Shewanella algae* | 0.230 ± 0.444 |
| *Shuttleworthia* | 1.970 ± 4.806 |
| *Solitalea* | 0.010 ± 0.035 |
| *Streptococcus anginosus* | 8.557 ± 19.041 |
| *Sutterella* | 0.250 ± 0.427 |
| *Thermaceae* | 0.270 ± 0.565 |
| *Thermales* | 0.250 ± 0.544 |
| *Thermus* | 1.400 ± 2.349 |
| *Ureaplasma* | 0.040 ± 0.181 |
| *Zoogloea* | 0.000 ± 0.002 |
