# Supplementary material for: Moderation effects of food intake on the relationship between urinary microbiota and urinary interleukin-8 in female type 2 diabetic patients
Source: PeerJ. 2020 Jan 28;8:e8481. doi: 10.7717/peerj.8481 (PMC6993747; doi:10.7717/peerj.8481)
Supplement: Supplemental Information 8 [file peerj-08-8481-s008.pdf]

Table S3 Correlation between urinary microbiota and IL-8 level

Taxon	r-value	p-value
Acinetobacter	-0.18	0.13
Acinetobacter rhizosphaerae	-0.07	0.59
Acinetobacter schindleri	0.2	0.11
Actinomyces	0.19	0.11
Akkermansia	-0.14	0.26
Akkermansia muciniphila	0.07	0.58
Anaerotruncus	0.28	0.02
Aquaspirillum	-0.12	0.34
Bacteroides	0.11	0.36
Bacteroides coprophilus	-0.01	0.91
Bacteroides ovatus	-0.01	0.93
Bacteroides uniformis	0.07	0.57
Blautia producta	-0.03	0.78
Cloacibacterium	-0.26	0.03
Comamonas	0.3	0.01
Coprococcus	-0.08	0.51
Coprococcus eutactus	-0.15	0.21
Corynebacterium	-0.08	0.47
Cytophaga	0.31	0.01
Dokdonella	-0.1	0.43
Dysgonomonas	0.2	0.11
Eubacterium biforme	0.08	0.49
Exiguobacterium	0.07	0.59
Faecalibacterium	0.03	0.82
Faecalibacterium prausnitzii	-0.1	0.43
Gemella	0.11	0.35
Geobacillus	-0.06	0.63
Giesbergeria	0.24	0.04
Klebsiella	-0.08	0.51
Lactobacillus	-0.11	0.38
Lactobacillus iners	0	0.98
Limnohabitans	0.1	0.39
Luteibacter	0.27	0.02
Megamonas	-0.08	0.54
Meiothermus	0.28	0.02
Microbacterium	-0.05	0.7
Mobiluncus	-0.06	0.63
Parabacteroides	0.12	0.32
Parabacteroides distasonis	-0.1	0.41
Prevotella	0.14	0.24
Prevotella copri	-0.03	0.78
Prevotella stercorea	0.09	0.45

Providencia	0.28	0.02
Pseudomonas	-0.06	0.61
Ruminococcus	0.28	0.02
Ruminococcus gnavus	-0.08	0.52
Shewanell algae	-0.07	0.58
Shuttleworthia	-0.14	0.28
Solitalea	0.11	0.36
Streptococcus anginosus	0.06	0.62
Sutterella	0.07	0.55
Thermaceae	-0.1	0.43
Thermales	-0.09	0.46
Thermus	0.24	0.05
Ureaplasma	0.01	0.93
Zoogloea	-0.03	0.83

Pearson's correlation analysis was used.
