# Supplementary material for: Moderation effects of food intake on the relationship between urinary microbiota and urinary interleukin-8 in female type 2 diabetic patients
Source: PeerJ. 2020 Jan 28;8:e8481. doi: 10.7717/peerj.8481 (PMC6993747; doi:10.7717/peerj.8481)
Supplement: Supplemental Information 9 [file peerj-08-8481-s009.pdf]

Table S4 Moderating effect of water intake on the relationship between *Ruminococcus* and IL-8 level

Variables		Controlling effect					Main effect		Interaction effect
		**Age**	**BMI**	**FBG**	**MS**	**UGLU**	***Ruminococcus***	**Water**	***Ruminococcus* ×Water**
Step 1	β	0.28	0.05	-0.17	-0.09	0.09			
	t	2.18	0.43	-1.28	-0.69	0.70			
	p	0.03	0.67	0.21	0.49	0.49			
	ΔF			1.94					
	ΔR^2			0.13					
	p			0.10					
Step 2	β	0.26	-0.01	-0.26	-0.05	0.10	0.20	-0.28	
	t	2.19	-0.07	-1.99	-0.42	0.77	1.78	-2.37	
	p	0.03	0.95	0.05	0.68	0.45	0.08	0.02	
	ΔF						5.70		
	ΔR2						0.14		
	p						0.01		
Step 3	β	0.29	0.02	-0.26	-0.05	0.09	-0.07	-0.30	-0.39
	t	2.52	0.18	-2.13	-0.42	0.72	0.47	-2.67	-2.74
	p	0.01	0.86	0.04	0.68	0.48	0.64	0.01	0.01
	ΔF								7.51
	ΔR2								0.08
	p						0.01		

Abbreviations: FBG: fasting blood glucose; UGLU: urine glucose level; MS: menstrual status; BMI: body mass index
