# Supplementary material for: Moderation effects of food intake on the relationship between urinary microbiota and urinary interleukin-8 in female type 2 diabetic patients
Source: PeerJ. 2020 Jan 28;8:e8481. doi: 10.7717/peerj.8481 (PMC6993747; doi:10.7717/peerj.8481)
Supplement: Supplemental Information 10 [file peerj-08-8481-s010.pdf]

Table S5 Moderating effect of fiber intake on the relationship between *Ruminococcus* and IL-8 level

Variables		Controlling effect					Main effect		Interaction effect
		**Age**	**BMI**	**FBG**	**MS**	**UGLU**	***Ruminococcus***	**Fiber**	***Ruminococcus* × Fiber**
Step 1	β	0.28	0.05	-0.17	-0.09	0.09			
	t	2.18	0.43	-1.28	-0.69	0.70			
	p	0.03	0.67	0.21	0.49	0.49			
	ΔF			1.94					
	ΔR^2			0.13					
	p			0.10					
Step 2	β	0.27	0.01	-0.19	-0.01	0.12	0.35	0.17	
	t	2.33	0.07	-1.54	-0.10	1.00	3.13	1.53	
	p	0.02	0.94	0.13	0.92	0.32	0.00	0.13	
	ΔF						7.99		
	ΔR^2						0.18		
	p						0.00		
Step 3	β	0.23	0.01	-0.11	-0.01	0.04	0.27	-0.10	0.42
	t	2.02	0.07	-0.90	-0.07	0.29	2.41	-0.68	2.73
	p	0.05	0.94	0.37	0.95	0.77	0.02	0.50	0.01
	ΔF								7.44
	ΔR^2								0.08
	p								0.01

Abbreviations: FBG: fasting blood glucose; UGLU: urine glucose level; MS: menstrual status; BMI: body mass index
