# Supplementary material for: Moderation effects of food intake on the relationship between urinary microbiota and urinary interleukin-8 in female type 2 diabetic patients
Source: PeerJ. 2020 Jan 28;8:e8481. doi: 10.7717/peerj.8481 (PMC6993747; doi:10.7717/peerj.8481)
Supplement: Supplemental Information 11 [file peerj-08-8481-s011.pdf]

Table S6 Moderating effect of Vit B3 intake on the relationship between *Ruminococcus* and IL-8 level

Variables		Controlling effect					Main effect		Interaction effect
		**Age**	**BMI**	**FBG**	**MS**	**UGLU**	***Ruminococcus***	**Vit B3**	***Ruminococcus*×Vit B3**
Step 1	β	0.28	0.05	-0.17	-0.09	0.09			
	t	2.18	0.43	-1.28	-0.69	0.70			
	p	0.03	0.67	0.21	0.49	0.49			
	ΔF			1.94					
	ΔR^2			0.13					
	p			1.00					
Step 2	β	0.21	-0.01	-0.17	-0.08	0.09	0.17	0.40	
	t	1.87	-0.06	-1.43	-0.66	0.73	1.55	3.67	
	p	0.07	0.95	0.16	0.51	0.47	0.13	0.00	
	ΔF						9.97		
	ΔR^2						0.21		
	p						0.00		
Step 3	β	0.18	-0.01	-0.07	-0.07	0.05	0.01	0.37	0.32
	t	1.64	-0.08	-0.60	-0.62	0.46	0.08	3.48	2.58
	p	0.11	0.94	0.55	0.54	0.65	0.94	0.00	0.01
	ΔF								6.67
	ΔR^2								0.07
	p								0.01

Abbreviations: FBG: fasting blood glucose; UGLU: urine glucose level; MS: menstrual status; BMI: body mass index
