# Supplementary material for: Moderation effects of food intake on the relationship between urinary microbiota and urinary interleukin-8 in female type 2 diabetic patients
Source: PeerJ. 2020 Jan 28;8:e8481. doi: 10.7717/peerj.8481 (PMC6993747; doi:10.7717/peerj.8481)
Supplement: Supplemental Information 12 [file peerj-08-8481-s012.pdf]

Table S7 Moderating effect of Vit E intake on the relationship between *Ruminococcus* and IL-8 level

Variables		Controlling effect					Main effect		Interaction effect
		**Age**	**BMI**	**FBG**	**MS**	**UGLU**	***Ruminococcus***	**Vit E**	***Ruminococcus* ×Vit E**
Step 1	β	0.28	0.05	-0.17	-0.09	0.09			
	t	2.18	0.43	-1.28	-0.69	0.70			
	p	0.03	0.67	0.21	0.49	0.49			
	ΔF			1.94					
	ΔR^2			0.13					
	p			0.10					
Step 2	β	0.23	-0.02	-0.24	-0.04	0.14	0.28	0.34	
	t	1.92	-0.17	-1.96	-0.36	1.11	2.57	3.06	
	p	0.06	0.86	0.06	0.72	0.27	0.01		
	ΔF						7.75		
	ΔR^2						0.17		
	p						0.00		
Step 3	β	0.21	-0.06	-0.25	-0.03	0.13	0.53	0.29	0.34
	t	1.84	-0.56	-2.10	-0.27	1.06	3.39	2.70	2.17
	p	0.07	0.58	0.04	0.79	0.30	0.00	0.01	0.03
	ΔF								4.69
	ΔR^2								0.05
	p								0.03

Abbreviations: FBG: fasting blood glucose; UGLU: urine glucose level; MS: menstrual status; BMI: body mass index
