# Supplementary material for: Moderation effects of food intake on the relationship between urinary microbiota and urinary interleukin-8 in female type 2 diabetic patients
Source: PeerJ. 2020 Jan 28;8:e8481. doi: 10.7717/peerj.8481 (PMC6993747; doi:10.7717/peerj.8481)
Supplement: Supplemental Information 15 [file peerj-08-8481-s015.docx]

**Table S10 Moderating effect of cholesterol intake on the relationship between *Comamonas* IL-8 level**

| **Variables** | | **Controlling effect** | | | | | **Main effect** | | **Interaction effect** |
| --- | --- | --- | --- | --- | --- | --- | --- | --- | --- |
|  |  | **Age** | **BMI** | **FBG** | **MS** | **UGLU** | ***Comamonas*** | **Cholesterol** | ***Comamonas* × Cholesterol** |
| Step 1 | β | 0.28 | 0.05 | -0.17 | -0.09 | 0.09 |  |  |  |
|  | t | 2.18 | 0.43 | -1.28 | -0.69 | 0.70 |  |  |  |
|  | p | 0.03 | 0.67 | 0.21 | 0.49 | 0.49 |  |  |  |
|  | ΔF |  |  | 1.94 |  |  |  |  |  |
|  | ΔR^2^ |  |  | 0.13 |  |  |  |  |  |
|  | p |  |  | 0.10 |  |  |  |  |  |
|  |  |  |  |  |  |  |  |  |  |
| Step 2 | β | 0.26 | 0.03 | -0.12 | -0.09 | 0.16 | 0.18 | 0.24 |  |
|  | t | 2.12 | 0.24 | -0.90 | -0.68 | 1.21 | 1.47 | 1.79 |  |
|  | p | 0.04 | 0.81 | 0.37 | 0.50 | 0.23 | 0.15 | 0.08 |  |
|  | ΔF |  |  |  |  |  | 4.39 |  |  |
|  | ΔR^2^ |  |  |  |  |  | 0.11 |  |  |
|  | p |  |  |  |  |  | 0.02 |  |  |
|  |  |  |  |  |  |  |  |  |  |
| Step 3 | β | 0.22 | 0.01 | -0.21 | -0.11 | 0.14 | -0.40 | 0.13 | 0.68 |
|  | t | 1.88 | 0.05 | -1.64 | -0.92 | 1.09 | -1.55 | 0.95 | 2.52 |
|  | p | 0.07 | 0.96 | 0.11 | 0.36 | 0.28 | 0.13 | 0.34 | 0.02 |
|  | ΔF |  |  |  |  |  |  |  | 6.33 |
|  | ΔR^2^ |  |  |  |  |  |  |  | 0.07 |
|  | p |  |  |  |  |  |  |  | 0.02 |

Abbreviations: FBG: fasting blood glucose; UGLU: urine glucose level; MS: menstrual status; BMI: body mass index
